# Daily fatigue-recovery balance monitoring with heart rate variability in well-trained female cyclists on the Tour de France circuit

**DOI:** 10.1371/journal.pone.0213472

**Published:** 2019-03-07

**Authors:** Anna Barrero, Frédéric Schnell, Guy Carrault, Gaelle Kervio, David Matelot, François Carré, Solène Le Douairon Lahaye

**Affiliations:** 1 M2S Laboratory, University of Rennes 2, Rennes, France; 2 CIC-CIT INSERM UMR 1099, Rennes, France; 3 University of Rennes 1, Department of Sports Medicine University Hospital of Rennes, INSERM, LTSI-UMR1099, Rennes, France; University of L’Aquila, ITALY

## Abstract

**Objectives:**

This study aimed to analyze the daily heart rate variability (HRV) in well-trained female cyclists during the 2017 Tour de France circuit and to relate it to the load and perceived exertion response.

**Methods:**

Ten female cyclists volunteered to participate in the study. HRV was recorded with a portable heart rate monitor each morning at rest in supine (7 min.) and upright (7 min.) positions, as well as throughout each day’s stage. Pre-Tour baseline HRV recordings were made, as well as during the four weeks following completion of the Tour. Exercise daily load was assessed using the training impulse score (TRIMPS). Post-exercise rate of perceived exertion (RPE) was assessed daily using the Borg CR-10 scale.

**Results:**

The results show a HRV imbalance, increase of sympathetic and decrease of vagal activities respectively, along the event that correlated with rate of perceived exertion (r = 0.46), training impulse score (r = 0.60), and kilometers (r = 046). The greatest change in HRV balance was observed the days after the greatest relative physical load. Mean heart rate and heart rate variability values returned to their baseline values one week after completion of the event.

**Conclusions:**

Despite incomplete recovery from day-to-day, fatigue is not summative or augmented with each successive stage and its physical load. Just one week is sufficient to restore baseline values. Heart rate and HRV can be used as a tool to strategically plan the effort of female cyclists that participate in multi-stage events.

## Introduction

Intensive endurance training is very demanding for the human organism and its regulatory systems [1]. Among them, cardiovascular control by the autonomic nervous system (ANS) presents alterations varying with the training versus recovery balance. Heart rate variability (HRV) represents sinus node modulation by the sympathetic and parasympathetic branches of the ANS [2]. HRV analysis is validated as a noninvasive method to study individual functional adaptations occurring to a given training stimulus in athletes [1,3].

HRV analysis is proposed as a valuable tool to study the athlete’s training versus recovery equilibrium and to detect early overreaching state that can decrease the athlete’s performance level [2, 3]. To our knowledge, most studies performed on this topic have focused on male athletes or mixed populations, and very few have studied female athletes. This omission is significant because the neural control of circulation differs with sex, especially before menopause [4]. HRV markers of sympathetic activity response after an orthostatic challenge test have been reported to be higher in male than in female athletes presented with a similar training load [5].

Moreover, HRV analysis in athletes has been focused mainly on the changes observed after acute post-exercise or throughout a training season in order to prevent fatigue and overtraining [1, 6–8]. HRV parameters changes induced by repeated days of endurance exercise have been scarcely studied, and only in male athletes [9]. Yet, it seems that the daily monitoring of HRV throughout a multi-day sporting event would be of interest for coaches to anticipate fatigue and to guide the athlete for his best final performance.

Therefore, the two objectives of this study were to describe a comprehensive characterization of resting heart rate (HR) and HRV changes in well-trained female cyclists during and following a multi-stage cycling event, and to propose an adapted method to follow fatigue and recovery in these athletes throughout this kind of sports event. We hypothesized that cyclists performing a Tour de France would incur a substantial HR increase and HRV decrease at rest along the days which would be worsened throughout the stages.

## Materials and methods

### Population

Ten healthy and well-trained (regional or national level) female cyclists coming from 4 countries (Belgium, France, Spain, Ukraine) completed the 21 stages of the men’s 2017 Tour de France, but one day before each stage of the official race. The event was performed without competition spirit. The cyclists completed the flat stages as a group, and each performed the time-trial and mountain stages at her own pace.

All participants gave informed written consent to participate in this study, which received the approval of the Rennes University Hospital Ethics Committee (Number 2013-A01524-41) and was conducted in accordance with the Declaration of Helsinki.

### Pre-participation medical evaluation

All athletes had a medical examination before the event, including a physical exam, a resting electrocardiogram (ECG), a transthoracic echocardiogram, and an incremental maximal cardiopulmonary exercise test on an electronically braked cycle ergometer (Excalibur Sport, Lode, The Netherlands). The exercise protocol started with a warm-up period (100 W for 5 min and 150 W for 1 min) followed by a step load-increase of 25 W/min until exhaustion. None of the athletes ingested any contraceptive and their menstrual cycle was not controlled.

### Description of the cycling event

The cycling event consisted of 21 stages and two rest days (Table 1). Due to the length of the stages, cyclists fed and hydrated continuously on the bike, according their needs. So individual food and fluid intake could not be controlled nor recorded during the stage nor between stages.

**Table 1:** Characteristics of the 2017 Tour de France stages.

Stage	Distance (km)	Profile
1	14	Time Trial
2	203.5	Flat stage
3	212.5	Medium mountain stage
4	207.5	Flat stage
5	160.5	Medium mountain stage
6	216	Flat stage
7	213.5	Flat stage
8	187.5	Medium mountain stage
9	181.5	High mountain stage
Rest 1		Rest Day
10	178	Flat stage
11	203.5	Flat stage
12	214.5	High mountain stage
13	101	High mountain stage
14	181.5	Medium mountain stage
15	189.5	Medium mountain stage
Rest 2		Rest Day
16	165	Medium mountain stage
17	183	High mountain stage
18	179.5	High mountain stage
19	222.5	Flat stage
20	22.5	Time trial
21	103	Flat stage

### HRV study

Baseline HRV values were established from the average of four days before the first stage. HRV was monitored each day during the event and on the first day following completion of the Tour, then once per week during the four weeks post-Tour. Cyclists refrained from any intense efforts during both the pre- and post-Tour periods.

#### Recording RR samples

The RR interval samples were recorded with a sampling rate of 1000 Hz with a HR monitor (Polar V800, Kempele, Finland) [10, 11]. Recordings were performed right after the cyclist woke up in the morning following an overnight fast, in a quiet, semi-darkened room, temperature range of 22–25°C. [2]. The RR samples were collected during two successive 7- minute periods, in supine and standing positions [1]. All athletes were familiarized with the monitor use.

#### HRV analysis

The RR data recorded was downloaded via Polar FlowSync software for Mac version 2.6.4 (Polar, Kempele, Finland) and exported for analysis with the Kubios HRV Standard software v3.0.0 2 (Biosignal Analysis and Medical Imaging Group at the Department of Applied Physics, Kuopio, Finland) [10]. For the analysis, the last 5 minutes window for each position was used. All the ectopic beats were filtered with the artifact correction option of the software. A very low threshold was applied when needed. Both time and frequency domain analyses were performed [1]. The root-mean-square difference of successive normal RR intervals (RMSSD), which reflects HR vagal modulation, was calculated. The high (HF:0.15–0.40 Hz) and low (LF:0.04–0.15Hz) frequency domains were analyzed. The HF band reflects vagal modulation while the LF band indicates both sympathetic and parasympathetic influences []. RMSSD, HFnu, LFnu (normal units) absolute values and their difference between supine and standing positions were calculated. The normalized (or normalized unit) spectral indices are defined by the developers of the Kubios HRV Standard software v3.0.0 2 as HFnu = HF / (LF + HF) and LFnu = LF / (LF + HF) (Biosignal Analysis and Medical Imaging Group at the Department of Applied Physics, Kuopio, Finland) in accordance with the recommendations [2].

### Load of exercise analysis

Each individual exercise daily load was calculated using the training impulse score (TRIMPS) method [12]. HR and GPS data were monitored continuously during each stage with the HR monitor. HR was divided into five zones, i.e. 50–60%, 60–70%, 70–80%, 80–90% and 90–100% of the individual maximal HR. Work load quantification was derived from the duration spent within the five HR zones [12].

### Rate of perceived exertion

The individual rate of perceived exertion (RPE) was evaluated with the Borg CR-10 scale [13] within 30 minutes after the end of each stage.

### Statistical analysis

Data are presented as mean ± SD. All analyses were performed using SPSS v.21 for Mac and STATISTICA v.7.1 for Windows. Normal Gaussian distribution of the data was verified by the Shapiro-Wilk test. Analysis of variance for repeated measurements was used. A Tukey’s post-hoc test was used to identify where the differences lie. In addition to the day-to-day effects on HR and HRV, the Tour was divided into 3 periods: period 1 (stages 1–9), period 2 (stages 10–15) and period 3 (stages 16–21), with rest days following stages 9 and 15.

Pearson’s product moment coefficient was calculated to assess the relationships among RPE, TRIMPS, the mileage of the stages, and the HR and HRV parameters. Significance was set at *P*<0.05.

## Results

All cyclists successfully finished the Tour de France.

Table 2 shows the demographic characteristics and the result of the cardiopulmonary exercise test of the subjects.

**Table 2:** Characteristics of the subjects.

	Weight	Height	BMI	Age	Previous cycling experience	Training	VO_2_ max	Maximal Power Output	Maximal power output relative
	(kg)	(m)	(kg.m^-2^)	(years)	(years)	(Km.week^-1^)	(ml.min.kg^-1^)	(Watt)	(W.kg^-1^)
**Subject 1**	55	1.69	19.3	32	18	250	55.5	325	5.91
**Subject 2**	57	1.64	21.2	28	6	200	59.2	300	5.26
**Subject 3**	52	1.45	24.7	31	17	200	55.5	280	5.38
**Subject 4**	63	1.68	22.3	30	11	225	49.7	300	4.76
**Subject 5**	52	1.6	20.3	32	7	200	51.8	275	5.29
**Subject 6**	59	1.7	20.4	26	17	100	55.6	275	4.66
**Subject 7**	72	1.7	24.9	29	6	200	42	300	4.17
**Subject 8**	50	1.62	19.1	37	28	100	60.1	275	5.5
**Subject 9**	52	1.57	21.1	29	2	250	57.4	275	5.29
**Subject 10**	58	1.68	20.6	43	28	150	49	250	4.31
**Mean**	**57**	**1.63**	**21.39**	**31.7**	**14**	**187.5**	**53.58**	**285.5**	**5.05**
±**SD**	**6.28**	**0.07**	**1.91**	**4.69**	**8.69**	**51.54**	**5.23**	**19.18**	**0.53**

$\dot{V}{\text{O}}_{2}$ max: maximal oxygen uptake, BMI: body mass index, SD: Standard deviation

HR, HRV, and workload values are presented for each stage (including resting days) of the cycling event in Table 3; and for each period in Table 4.

**Table 3:** Mean values (±SD) by stage for 10 female cyclists during the 2017 Tour de France.

	Resting HR Supine	Resting HR Standing	Resting Heart Rate Standing-supine	RMSSD Supine	RMSSD Standing	RMSSD Supine-Standing	HF nu Supine	HF nu Standing	HF nu Supine-Standing	LF nu Supine	LF nu Standing	LF nu Standing-supine	Mean exercise HR	TRIMP	RPE
Stage	(bpm)	(bpm)	(bpm)	(ms)	(ms)	(ms)	(n.u.)	(n.u.)	(n.u.)	(n.u.)	(n.u.)	(n.u.)	(bpm)	(n.u.)	(n.u.)
*Baseline*	51.91 (6.12)	76.80 (7.32)	24.89 (4.77)	93.46 (33.24)	25.97 (12.63)	67.49 (26.00)	56.92 (17.69)	12.26 (7.53)	44.66 (17.64)	43.03 (17.72)	87.72 (7.53)	44.69 (17.67)			
1	57.47 (9.15)	83.05 (9.32)	25.58 (4.59)	59.53 (36.24)	10.42 (4.90)	49.11 (18.63)	53.28 (21.22)	7.01 (4.57)	46.27 (10.91)	46.63 (21.29)	92.98 (4.57)	46.35 (10.95)	113.44 (11.09)	102 (26)	1.20 (0.42)
2	58.38 (8.19)	82.34 (14.05)	23.96 (10.17)	70.67 (31.06)	17.75 (10.48)	52.92 (21.44)	43.13 (15.94)	12.35 (12.99)	30.78 (16.72)	56.78 (15.94)	87.64 (12.99)	30.86 (16.75)	127.90 (10.13)	1202 (210)	4.00 (0.82)
3	69.01 (7.32)	88.77 (8.11)	19.76 (5.45)	32.30 (9.48)	15.07 (7.10)	17.23 (5.52)	29.26 (7.99)	7.69 (5.91)	21.57 (5.32)	70.69 (8.01)	92.30 (5.91)	21.61 (5.29)	130.80 (8.40)	1420 (236)	5.40 (1.17)
4	72.55 (12.15)	82.56 (11.57)	10.01 (5.59)	35.02 (19.01)	25.03 (16.75)	9.99 (10.81)	25.05 (11.18)	8.68 (4.89)	16.38 (8.91)	74.90 (11.17)	91.30 (4.90)	16.40 (8.90)	125.90 (10.14)	1200 (287)	4.90 (1.66)
5	69.05 (10.96)	78.38 (8.13)	9.33 (6.77)	40.78 (20.04)	28.65 (21.35)	12.13 (24.14)	33.24 (16.74)	15.76 (11.61)	17.47 (12.27)	66.70 (16.77)	84.18 (11.63)	17.48 (12.28)	128.60 (9.11)	995 (189)	6.50 (1.43)
6	65.59 (12.14)	72.95 (10.91)	7.36 (7.98)	53.57 (27.16)	37.71 (25.19)	15.86 (20.18)	35.96 (15.29)	26.58 (14.04)	9.38 (8.51)	63.96 (15.32)	73.36 (14.06)	9.40 (8.53)	122.40 (6.57)	1193 (200)	5.50 (1.18)
7	64.23 (11.04)	78.47 (11.46)	14.24 (6.42)	58.19 (29.82)	32.51 (19.83)	25.68 (17.13)	44.87 (16.72)	17.30 (8.69)	27.57 (14.32)	55.07 (16.74)	82.65 (8.71)	27.58 (14.34)	124.20 (7.83)	1115 (255)	5.30 (1.70)
8	64.75 (8.44)	76.52 (7.89)	11.77 (5.62)	56.35 (27.45)	29.14 (17.23)	27.20 (11.05)	32.72 (15.52)	17.76 (9.80)	14.96 (13.12)	67.22 (15.55)	82.22 (9.81)	14.99 (13.13)	131.33 (4.95)	1478 (227)	7.00 (1.56)
9	64.53 (7.69)	77.21 (9.30)	12.68 (6.92)	49.54 (20.76)	27.55 (18.21)	21.99 (18.32)	35.79 (13.17)	17.27 (9.92)	18.52 (13.70)	64.14 (13.18)	82.65 (9.95)	18.51 (13.75)	131.50 (6.66)	1804 (306)	8.50 (1.18)
*Rest day*	67.70 (7.75)	78.02 (8.66)	10.32 (8.79)	38.56 (13.19)	23.74 (12.34)	14.82 (16.58)	43.05 (12.82)	19.88 (16.52)	23.17 (17.01)	56.77 (12.83)	80.08 (16.53)	23.31 (16.90)			
10	55.69 (7.97)	72.74 (7.99)	17.05 (8.70)	60.99 (30.50)	30.41 (20.34)	30.58 (26.92)	45.87 (21.32)	19.81 (11.95)	26.07 (17.18)	54.07 (21.34)	80.16 (11.96)	26.09 (17.18)	110.70 (15.27)	776 (125)	2.11 (1.17)
11	58.96 (10.52)	74.10 (14.19)	15.13 (9.88)	55.10 (25.30)	19.60 (9.89)	35.50 (16.14)	46.62 (19.30)	24.98 (22.50)	21.64 (20.29)	53.29 (19.33)	74.64 (22.37)	21.35 (19.94)	113.38 (3.02)	785 (139)	3.56 (2.65)
12	58.13 (6.27)	77.38 (10.68)	19.25 (8.60)	51.24 (20.86)	27.28 (24.35)	23.97 (22.16)	41.16 (18.87)	16.39 (12.88)	24.77 (14.33)	58.79 (18.91)	83.59 (12.88)	24.80 (14.33)	121.88 (13.62)	1634 (243)	7.89 (1.17)
13	67.71 (5.36)	81.68 (9.22)	20.74 (22.05)	32.78 (12.01)	13.49 (5.99)	19.29 (7.02)	41.27 (10.53)	10.08 (3.75)	31.19 (8.45)	58.66 (10.54)	89.89 (3.77)	31.23 (8.48)	120.40 (12.29)	787 (78)	4.90 (0.74)
14	60.25 (7.17)	73.76 (10.50)	13.51 (4.92)	62.61 (23.12)	28.46 (14.22)	34.15 (18.87)	45.76 (16.71)	25.44 (16.47)	20.32 (14.04)	54.20 (16.72)	74.48 (16.45)	20.28 (14.04)	114.90 (5.00)	971 (167)	4.80 (1.62)
15	58.30 (8.69)	74.80 (9.66)	16.50 (8.82)	65.59 (23.40)	25.37 (12.28)	40.22 (19.98)	39.09 (14.15)	22.41 (15.51)	16.68 (21.18)	60.84 (14.19)	77.51 (15.54)	16.67 (21.20)	114.11 (15.95)	1303 (183)	6.90 (1.66)
*Rest day*	59.53 (6.35)	77.81 (8.22)	18.28 (5.44)	51.61 (12.93)	21.88 (10.39)	29.73 (10.83)	42.43 (13.42)	16.15 (10.03)	26.28 (12.78)	57.40 (13.47)	83.76 (9.96)	26.35 (12.71)			
16	54.22 (6.69)	77.10 (13.81)	22.88 (7.68)	70.59 (32.57)	23.49 (16.87)	47.10 (25.92)	60.11 (22.43)	14.21 (12.81)	45.90 (16.57)	39.85 (22.44)	85.76 (12.82)	45.91 (16.59)	123.80 (9.03)	963 (179)	4.78 (2.17)
17	55.71 (8.42)	74.13 (10.50)	18.42 (8.15)	70.89 (20.89)	29.49 (17.33)	41.40 (23.85)	48.17 (19.05)	20.33 (20.26)	27.84 (13.94)	51.78 (19.06)	79.64 (20.26)	27.86 (13.96)	126.33 (11.88)	1698 (248)	7.22 (1.30)
18	63.95 (11.94)	82.34 (11.47)	18.39 (8.72)	52.18 (18.99)	18.16 (9.19)	34.02 (17.78)	40.65 (18.90)	13.73 (9.06)	26.92 (12.35)	59.31 (18.91)	86.24 (9.06)	26.93 (12.35)	117.50 (11.72)	1267 (193)	6.60 (1.35)
19	56.47 (6.17)	77.33 (9.82)	20.86 (7.14)	57.78 (23.74)	19.49 (10.72)	38.30 (18.36)	48.94 (19.03)	12.44 (7.03)	36.51 (17.43)	51.02 (19.03)	87.52 (7.06)	36.51 (17.45)	110.13 (10.06)	1245 (242)	6.00 (2.16)
20	60.47 (6.65)	72.63 (8.18)	12.16 (4.50)	57.30 (17.56)	25.60 (6.58)	31.70 (13.61)	34.30 (16.91)	21.62 (10.23)	12.68 (17.83)	65.66 (16.93)	78.31 (1.22)	12.65 (17.86)	97.43 (5.41)	124 (9)	2.30 (2.36)
21	59.18 (14.16)	72.67 (11.13)	13.49 (2.27)	46.33 (17.19)	20.39 (13.17)	25.93 (3.79)	23.81 (13.73)	10.53 (4.21)	13.28 (6.61)	76.16 (13.73)	89.43 (4.19)	20.89 (24.98)	98.40 (7.16)	276 (46)	1.40 (0.97)
*Day 1 post event*	56.34 (7.55)	78.35 (12.03)	22.00 (4.88)	50.30 (16.46)	18.60 (8.44)	31.69 (6.26)	38.67 (16.99)	8.96 (6.79)	29.71 (13.13)	61.30 (17.01)	91.02 (6.80)	29.72 (13.14)			

Data are presented as mean (±SD)

HR: heart rate, TRIMP: training impulse, RPE: rate of perceived exertion, n.u.: normalized units, bpm: beats per minute.

**Fig 1:**
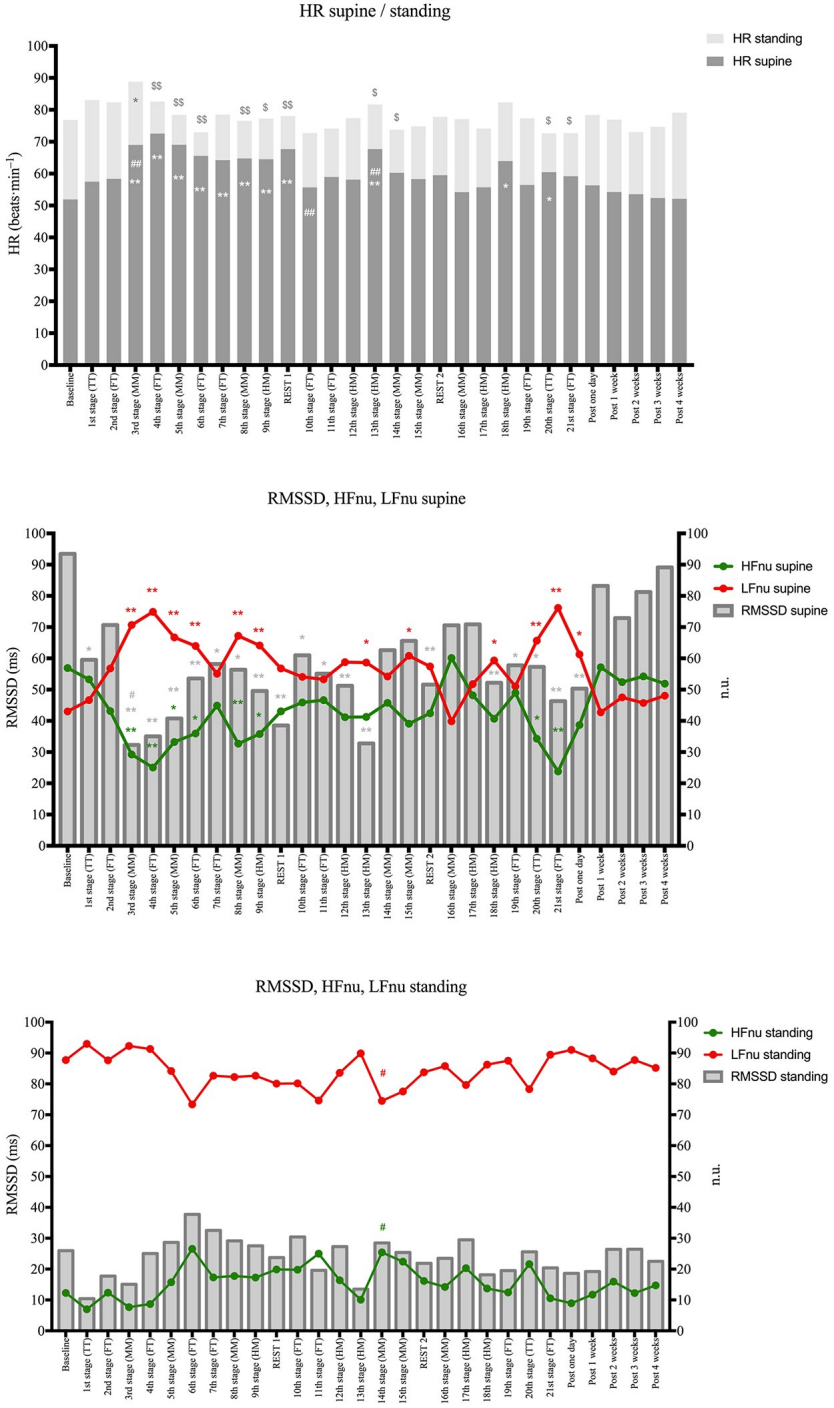
Evolution of Heart rate and HRV parameters stage by stage. Data are presented as mean group values. Absolute HR values different from baseline value* (*P* < 0.05); different from previous stage # (*P* < 0.05). HR standing- HR supine values different from baseline value $ (*P* < 0.05). When reading the differences, note that all the values were recorded on the morning of each stage, which reflects the load effect of the previous stage. HR: heart rate; RMSSD: root mean square of successive differences; HF: high frequency; LF: low frequency; ms: milliseconds, n.u.: normalized units; TT: time trial; FT: flat stage; MM: medium mountain stage; HM: high mountain stage; Post: post-cycling event period.

**Fig 2:**
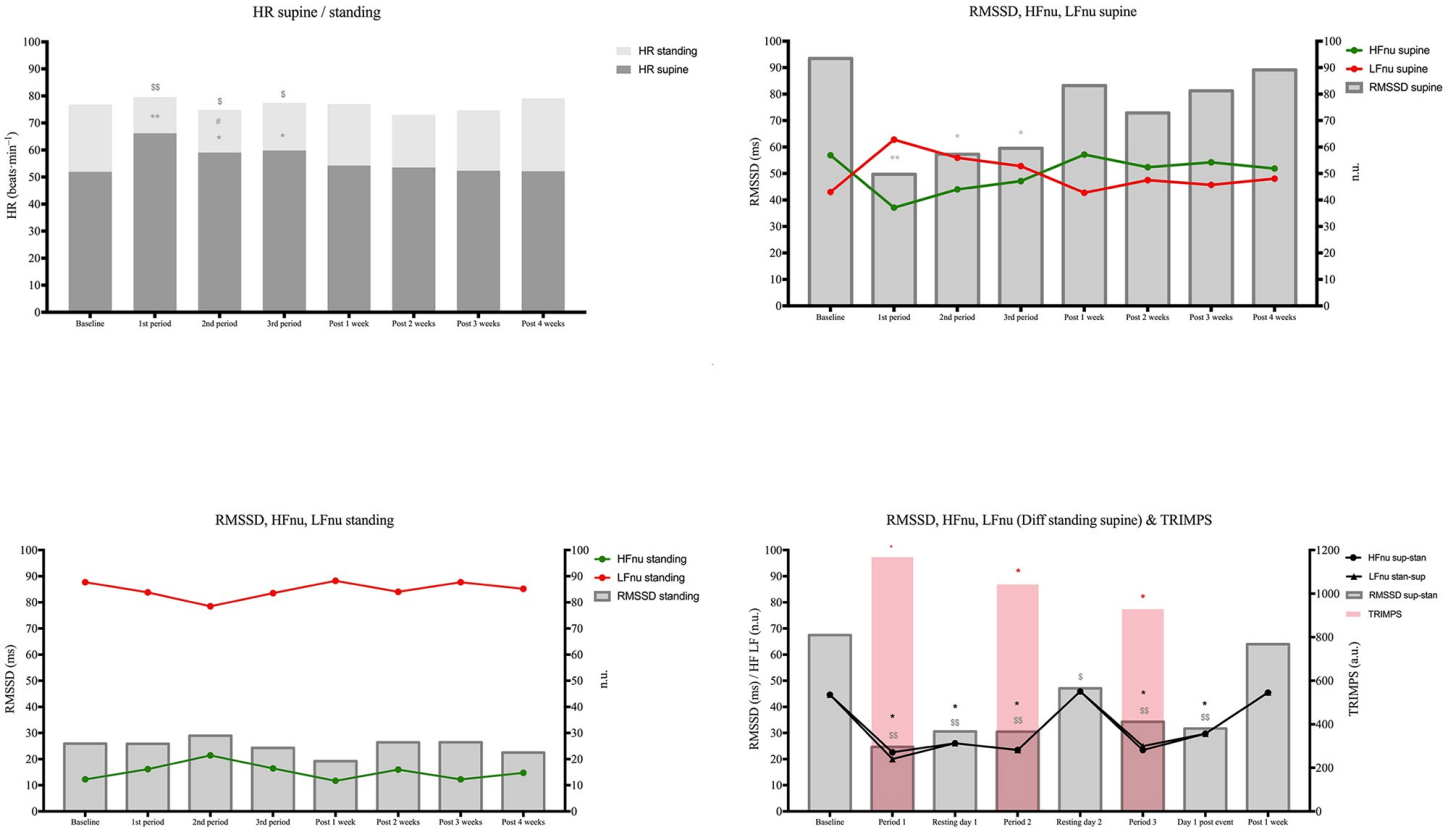
Evolution of heart rate and HRV parameters by period. Data are presented as mean group values for each period. Period 1: from stage 1 until stage 9 inclusive, period 2 from stage 10 until stage 15 inclusive, period 3 from stage 16 until stage 21 inclusive. Post: post-Tour period. Statistical differences: Different from baseline * (*P* < 0.05); different from the previous period # (*P* < 0.05). Standing-supine values different from baseline $ (*P* < 0.05); different from previous period £ (*P* < 0.05). HR: heart rate; RMSSD: root mean square of the successive differences; HF: high frequency; LF: low frequency; ms: milliseconds; n.u.: normalized units; TRIMPS: training impulse.

To avoid making the table too complicated the significant differences of the relevant parameters are illustrated in Figs 1 and 2.

**Table 4:** Mean values (±SD) by period for 10 female cyclists during the 2017 Tour de France.

	Supine HR	Standing HR	Standing-supine HR	Supine RMSSD	Standing RMSSD	Supine-standing RMSSD	Supine HF nu	Standing HF nu	Supine-standing HF nu	Supine LF nu	Standing LF nu	Standing-supine LF nu	TRIMP	RPE
Phases	(bpm)	(bpm)	(bpm)	(ms)	(ms)	(ms)	(n.u)	(n.u.)	(n.u.)	(n.u.)	(n.u.)	(n.u.)	(n.u.)	(n.u.)
*Baseline*	51.91 (6.12)	76.80 (7.32)	24.89 (4.77)	93.46 (33.24)	25.97 (12.63)	67.49 (26.00)	56.92 (17.69)	12.26 (7.53)	44.66 (17.64)	43.03 (17.72)	87.72 (7.53)	44.69 (17.67)		
*Period 1*	66.23 (8.60)	79.60 (8.36)	15.15 (4.38)	49.68 (13.66)	25.85 (13.50)	24.69 (5.56)	37.14 (12.19)	16.15 (8.21)	22.61 (6.43)	62.79 (12.21)	83.81 (8.21)	20.02 (6.42)	1167.67 (465.83)	5.37 (2.04)
*Rest day 1*	55.69 (7.98)	72.74 (7.99)	17.05 (8.70)	60.99 (30.50)	30.41 (20.34)	30.58 (26.92)	45.87 (21.32)	19.81 (11.95)	26.07 (17.18)	54.07 (21.34)	80.16 (11.96)	26.09 (17.18)		
*Period 2*	59.76 (6.33)	75.24 (9.86)	17.24 (6.55)	56.25 (14.52)	27.85 (16.94)	30.48 (9.18)	43.68 (13.05)	22.00 (13.62)	23.48 (10.13)	56.25 (13.08)	77.90 (13.57)	23.45 (10.05)	1042.67 (353.68)	5.03 (2.12)
*Rest day 2*	54.22 (6.70)	77.10 (13.81)	22.88 (7.68)	70.59 (32.57)	23.49 (16.87)	47.10 (25.92)	60.11 (22.43)	14.21 (12.81)	45.90 (16.57)	39.85 (22.44)	85.76 (12.82)	45.91 (16.59)		
*Period 3*	60.63 (9.46)	76.96 (9.87)	16.66 (5.07)	57.20 (16.75)	24.38 (11.31)	34.27 (12.03)	43.95 (15.02)	17.33 (12.60)	23.45 (10.40)	56.00 (15.03)	82.63 (12.59)	24.97 (12.48)	928.83 (613.36)	4.72 (2.38)
*Day 1 post event*	56.34 (7.55)	78.35 (12.03)	22.00 (4.88)	41.91 (25.27)	18.61 (8.44)	31.69 (6.26)	38.67 (16.99)	8.96 (6.79)	29.71 (13.13)	61.30 (17.01)	91.02 (6.80)	29.72 (13.14)		

Data are presented as mean (SD). Period 1: from stage 1 until stage 9 included, period 2 from stage 10 until stage 15 included, period 3 from stage 16 until stage 21 included.

HR: heart rate, TRIMP: training impulse, RPE: rate of perceived exertion, n.u.: normalized units, bpm: beats per minute.

In order to see the evolution of the subjects along the cycling event, Fig 1 presents HR, HRV, and workload values for each stage (including resting days).

To have a greater vision of the subjects’ evolution, Fig 2 illustrates HR and HRV parameters by periods.

### Heart rate evolution

Supine HR during period 1 increased in comparison with its basal value after stage 2 (flat). This increase persisted until stage 9 with no difference among the stages. During period 2, HR increased after the first medium mountain stage (12^th^ stage) and then decreased to its basal value. During period 3, HR was higher than its basal value only after the 17^th^ (high-mountain) and 19^th^ (flat) stages. For each of the three periods the global supine HR was higher than the basal value. HR was higher during period 1 than during periods 2 and 3, but no difference was observed between periods 2 and 3. The HR value returned to its basal values after each rest day and during the recovery period.

The standing HR values presented no difference during the cycling event, except after stage 2 (Fig 1). No differences were observed among the three periods. Positive correlations were observed between supine HR and the distance of the stage (r = 0.46, P = 0.037), TRIMPS (r = 0.60, P = 0.004), and RPE (r = 0.46, P = 0.038). The higher the workload, the higher supine HR was after a recovery night.

### HFnu and LFnu evolution

Supine HFnu was decreased compared to its basal value from stage 2 until the first rest day, after which it went back to the basal value (Fig 1). Then, the HFnu value decreased from the 19^th^ stage and returned to its basal value after the 21^st^ stage. Lastly, the HFnu value was very close to its basal value after one week of recovery. As expected, LFnu mirrored the evolution of HFnu, with an increase of LFnu in compared to basal and recovery values. The changes in daily LFnu were more frequently different than the HFnu ones and overall LFnu was higher than HFnu during the entire cycling event. The opposite was observed only before the event start and during the recovery. No differences were observed between mean supine HFnu and LFnu values by period (Fig 2). The daily standing-supine differences for HFnu and LFnu were lower than their basal values during the entire Tour, except after the second rest day. No differences were observed after one week of recovery (Fig 1). The mean period analysis of standing-supine difference of these parameters showed the same significant differences as the daily values compared to baseline during all three periods, except for the second rest day (Fig 2). The standing HFnu values were lower and LFnu values were higher than supine, with no variation during the cycling event (Figs 1 and 2).

### RMSSD evolution

The supine RMSSD global evolution showed the same trend as HFnu, even though more stages showed RMSSD declines (Fig 1). The same was observed with the periods analysis, with a more marked RMSSD decrease during all the periods in comparison to the basal and recovery periods (Fig 2). No significant variation was noted for the standing RMSSD.

The daily RMSSD standing-supine difference was lower than the basal value during the whole cycling period, except after the second rest day and after one week of recovery (Fig 2). There was a significant correlation between supine RMSSD and distance of the stages (r = -0.45, *P* = 0.0001). The mean period analysis of RMSSD standing-supine show a reduction compared to baseline but with no differences among periods (Fig 2).

### Rate of perceived exertion evolution

There was a more marked decrease after the first rest day than after the second one (Table 3). The RPE evolution was positively related to TRIMPS (r = 0.61, *P* = 0.003) and to the distance of the stages (r = 0.91, *P* = 0.0001).

## Discussion

To our knowledge, this study is the first to investigate the day-to-day resting HR and HRV responses in well-trained female cyclists during a multi-stage cycling event. We observed variable responses of HR and HRV parameters in relation to the distance of the stage throughout the Tour which partially confirms our hypothesis. The difference between standing and supine HR and HRV has a practical application for monitoring fatigue in a female cyclist.

### Evolution of HR and HRV indices during the cycling event

As proposed, we studied the HR standing/supine difference and HRV indices responses to the orthostatic stress that reflects the adaptation of the sinus node [1]. Because significant changes concerned mainly resting values with no change for standing values (Figs 1 and 2), the discussion will concern resting HRV indices. Globally we observed an inversion of the ratio LFnu/HFnu with a value higher than 1 throughout the event, except for one value after the second rest day, in comparison to a LFnu/HFnu lower than 1 during the pre-race and post-race periods. During the first period (stages 1–9) of the event, both HR and HRV changes show a biphasic pattern. Indeed, in comparison to the basal value, period 1 showed three parts. No change was observed after stages 1 and 2, then after stages 3 to 5 a statistically significant decrease of HR response was noted. Concerning HRV indices, we observed a significant increase in the LFnu/HFnu ratio explained by a significant increase in sympathetic (LFnu) input associated with a significant decrease in parasympathetic (HFnu) input. Lastly, in stages 6 to 9 there was a less marked decrease of HR associated with a progressive increase in HFnu and a decrease in LFnu (Figs 1 and 2). The decrease of HR standing/supine difference observed during stages 3 to 9 was due to both an increase of resting HR and a decrease of standing HR. Throughout the second and third periods the HR as HRV responses to orthostatic stress were attenuated, with similar changes as seen during stages 6–9. The decrease of HR responses during these two periods was due to a lesser increase in standing HR than during the basal period, with fewer changes in supine HR. It must be noted that despite a very low level of mean TRIMPS during the last two stages of the Tour (20–21), 124 au and 276 au, respectively, we observed a marked decrease of HR response to orthostatic stress due to a low increase in HR and a marked increase in LFnu/HFnu when standing. This discrepancy between TRIMPS and HR response could be interpreted as a fatigue sign.

The biphasic response observed during period 1 was not linked to a workload difference (mean TRIMPS 1205 au and 1397 au, respectively, for stages 3–5 and 6–9; NS). The HR and HRV response we observed could be explained by the change in the cardiac preload reported after a few days of intense training, due to delayed hormonal responses [14, 15]. A similar biphasic response observed in left ventricular function has been reported after a four-day simulated multi-stage cycling [16]. The higher workload recorded during the first period compared to the second and third periods could explain the differences observed in HR and HRV responses.

The beneficial effect of a rest day for the responses to orthostatic stress appears clearly in Figs 1 and 2, for one day as well as during the recovery period post-Tour. Concerning the effects of the rest days proposed during the race, to our knowledge only one study investigated the effects on HRV indices of rest days (days 10 and 17) during the Vuelta a España performed by male cyclists. Similar to the present study, no difference was observed between rest days and pre-race HRV indices [9]. Our results show that after one week of post-race recovery, all HR parameters and HRV indices were similar to the pre-event basal values.

Supine RMSSD values, another parasympathetic parameter, mimics the globally responses of HFnu (Figs 1 and 2). After the two hardest stages of the event (HM stages 9 and 12, with 181 and 214 km), an acute decrease of supine and standing RMSSD values was observed (Fig 1).

To summarize, in comparison to the basal value, the day-to-day HR and HRV responses to an orthostatic active test vary with the duration of the cycling event. The temporary marked alterations of HR responses and of autonomic balance (LFnu/HFnu) observed during the first stages do not seem to be due to a real fatigue state but to acute stress stimuli (subjects not used to these distances). During the next stages both a stable and modest decrease of HR responses and an increase of LFnu/HFnu were observed. We did not observe a pattern of fatigue accumulation with the repetition of stages. However, at the end of the event (two last stages) marked alteration of the parameters studied was again observed. These results are in accordance with the most common ‘fatigue’ pattern described in athletes during intensive training [17]. This is confirmed by the quick and complete recovery after one week. Another observation is that supine HR response correlated with the distance of the stages, TRIMPS, and RPE.

### Practical applications from the study

Several studies, reviews, and meta-analyses are in favor of the value of the HRV follow-up in male and female athletes to guide their training to prevent overreaching and overtraining [1, 3, 18, 19]. Our study corroborates that HRV is a useful non-invasive tool that can help to program training and identify fatigue in endurance cyclists. From a practical point of view, the results of this study can help to propose the more useful validated parameters for a daily practice during a multistage cycling event. Globally, given their lack of sensitivity the contribution of the isolated variations of the HR and HRV standing parameters used in this study appear as the less contributive (Figs 1 and 2). Concerning the HR survey, the difference between standing and supine HR after an active orthostatic test showed a good value and the variations of resting supine HR appear as a very simple tool. Concerning the HRV parameters, the use of two indices (RMSSD and HFnu) reflecting the response of the sinus node to the parasympathetic stimuli does not seem useful. The association of the two spectral indices LFnu and HFnu in supine position appears to be the most informative.

### Limitations of the study

This study presents some limitations. First, the number of athletes studied was small. This limitation was mainly due to the specific and heavy daily logistic associated with this project that was not an official competition. Second, the cycling event was not a competitive one and our results need to be confirmed in competition because of the specific somatic stress linked to competition. Third, the daily recovery included only one night’s rest without a scientific adapted nutrition or massage session. A more scientific recovery might alter the observed results.

## Conclusions

The characterization of the HRV response during the entire Tour de France adds new information concerning the fluctuating sympathovagal response of an ultra-endurance event. Despite incomplete recovery, the extent of cardiac suppression with each successive stage, and its physical load, is not summative or augmented. Just one week is enough to restore baseline values. These results suggest that well-trained female cyclists need only one week to recover from an effort such as the Tour de France circuit.
